# Active ingredients isolated from *Periplaneta americana* L. inhibit the inflammation of the colonic mucosa and regulate the gut microbiota in DSS-induced ulcerative colitis in mice

**DOI:** 10.3389/fphar.2025.1615989

**Published:** 2025-09-19

**Authors:** Yanhuan Wang, Fa Ni, Genrui Wu, Huai Xiao, Zhibin Yang, Chenggui Zhang, Hairong Zhao, Heng Liu

**Affiliations:** ^1^ Yunnan Provincial Key Laboratory of Entomological Biopharmaceutical R&D, College of Pharmacy, Dali University, Dali, China; ^2^ National-Local Joint Engineering Research Center of Entomoceutics, Dali University, Dali, China

**Keywords:** *Periplaneta americana* L., ulcerative colitis, cytokine, inflammatory response, gut microbiota

## Abstract

**Introduction:**

Ulcerative colitis (UC) is a chronic inflammatory bowel disease characterized by colonic mucosal inflammation, compromised intestinal barrier function, and gut microbiota dysbiosis. Current therapies often have significant limitations, including adverse effects, highlighting the need for safer alternatives. *Periplaneta americana* L. (PA), documented in Shennong's Herbal Classic, possesses anti-inflammatory, tissue-repairing, and immunomodulatory properties. While previous studies demonstrated efficacy of PA in rodent UC models generated using different inducers, the active components within aqueous extracts (PAW) and their comparative effects remain unclear.

**Materials:**

To address this gap, this study investigated the composition and therapeutic activity of PAW and its sequentially fractionated components based on molecular weight: PAW1 (< 3 kDa), PAW2 (3-10 kDa), and PAW3 (> 10 kDa) using membrane separation. Using a dextran sulfate sodium (DSS)-induced UC model in C57BL/6 mice, we compared the effects of unfractionated PA and its fractions (PAW1, PAW2, PAW3) on UC pathology and intestinal flora.

**Results:**

Our results demonstrate that PA, PAW1, PAW2, and PAW3 ameliorated key UC-associated pathologic features; notably, the unfractionated PA exhibited superior efficacy compared to its individual fractions. PA treatment significantly mitigated DSS-induced body weight loss, disease activity index scores, and colon shortening. It preserved intestinal mucosal integrity, evidenced by increased goblet cell numbers and elevated expression of tight junction proteins (occludin-1, ZO-1). PA treatment reduced colonic inflammation by significantly downregulating pro-inflammatory mediators (NF-κB-p65, TLR4, MyD88, TNF-α, IL-17A, IFN-γ, MPO, iNOS) and upregulating the anti-inflammatory cytokine IL-10, while IL-4 levels were also modulated. Furthermore, PA treatment attenuated intestinal dysbiosis in UC mice, characterized by an increase in beneficial bacteria (e.g., *Psychrobacter*) and a decrease in taxa like *Actinobacteriota*.

**Conclusion:**

These findings collectively indicate that the aqueous extract of *Periplaneta americana* L. and its fractions possess significant therapeutic potential for UC treatment, with the unfractionated extract showing the most pronounced benefits via modulating inflammation, restoring barrier function, and rebalancing gut microbiota.

## 1 Introduction

Ulcerative colitis (UC) is a chronic, nonspecific inflammatory bowel disease characterized by mucosal inflammation primarily affecting the colon and rectum (Matsuoka et al., 2018; Stange et al., 2008). Clinical manifestations include diarrhea, abdominal pain, and hematochezia (Liu et al., 2024). Although its etiology remains incompletely defined, UC pathogenesis involves genetic susceptibility, dysregulated immune responses, environmental triggers, and gut microbiota dysbiosis (Farrell and Peppercorn, 2002). Critically, immune hyperactivation disrupts the intestinal epithelial barrier and amplifies pro-inflammatory mediators (e.g., TNF-α, IL-6, IL-1β), driving sustained mucosal inflammation and tissue injury (Sun et al., 2024; Wang et al., 2024). Current therapeutic strategies—including aminosalicylates, glucocorticoids, immunosuppressants, and biologics (Long, 2024)—are limited by suboptimal efficacy in refractory cases and significant adverse effects (Aebisher et al., 2024). Consequently, novel therapies targeting multiple pathogenic pathways are urgently needed. Notably, gut dysbiosis, marked by reduced microbial diversity and expansion of pathobionts (e.g., *Proteobacteria*), is a hallmark of UC pathogenesis (Guo et al., 2020; Wu et al., 2019) Restoring microbial homeostasis thus represents a promising therapeutic avenue.

*Periplaneta americana* L. (*PA*), commonly known as the American cockroach, has a long history of medicinal use in ancient Chinese practices (Zhao et al., 2024). It is documented in classical texts such as *Shennong’s Herbal Classic* and has been traditionally utilized to treat conditions, including blood stasis, firmness and coldness symptoms, heat sensations, accumulations, and throat paralysis. Contemporary scientific investigations have revealed that PA is a rich source of bioactive compounds, including peptides, proteins, polysaccharides, amino acids, and trace elements (Chen et al., 2025; Liang et al., 2022). Notably, peptide constituents, including antimicrobial peptides and anti-inflammatory peptides, exhibit significant biological activities, such as anti-inflammatory, antioxidant, immunomodulatory, and tissue-repairing effects (Li et al., 2023a; Yoon et al., 2017). Given that UC pathogenesis is intricately linked to immune imbalance, excessive inflammation, oxidative stress, and gut microbiota dysbiosis (Wu et al., 2022a), bioactive peptides derived from PA may exert therapeutic effects on UC through multiple pathways.

While recent studies suggest PA extracts mitigate UC in preclinical models (Ma et al., 2018; Xie et al., 2024), critical knowledge gaps persist: (1) the key bioactive constituents within these extracts responsible for therapeutic efficacy have not been clearly identified; (2) the differential effects of fractions with distinct molecular weights, particularly their ability to target gut microbiota, remain underexplored. Thus, the present study isolates and characterizes PA aqueous extract (PAW) fractions by molecular weight (PAW1: <3 kDa; PAW2: 3–10 kDa; PAW3: >10 kDa), evaluates their comparative efficacy in a dextran sulfate sodium (DSS)-induced murine UC model, and investigates their effects on inflammation, barrier integrity, and gut microbiota restoration.

## 2 Materials and methods

### 2.1 Animals

A total of 42 4-week-old C57BL/6 male mice (18 ± 2 g) were obtained from Chengdu Dashuo Laboratory Animal Co., Ltd. [Chengdu, China; Certificate No SCXK (Chuan) 2020-0030]. Mice were housed in an animal laboratory with a temperature and humidity of 22 °C ± 2 °C and 50% ± 5%, respectively, and a 12 h light/dark cycle (Andersson et al., 2024). The mice received standard water and food *ad libitum*. All experimental procedures were approved by the Animal Ethics Committee of Dali University (Approval No. 2023-SL-274, Dali, China), and conducted in accordance with the ARRIVE guidelines.

### 2.2 Materials and reagents

PA (Lot. 20200521) was provided by Yunnan Jingxin Biotechnology Co., Ltd. (Yunnan, China) and authenticated by Yang Zizhong. Olsalazine sodium capsules (Lot. 210206) were procured from Zhejiang Zhongyi Pharmaceutical Co., Ltd. (Zhejiang, China). DSS (Lot. 1132-222) was obtained from Shenzhen Lijing Biochemistry Technology Co., Ltd. (Shenzhen, China). Anhydrous ethanol (Lot. 1703113601) and formic acid (FA, Lot. 20171122) were acquired from Fuchen Chemical Reagent Co., Ltd. (Tianjin, China) and Tianjin Windship Chemical Reagent Technology Co., Ltd. (Tianjin, China), respectively. Acetonitrile (ACN, HPLC grade, Lot. 204127) was sourced from Fisher Scientific (Fair Lawn, NJ, United States). A fecal occult blood test kit (Lot. 20230520) was purchased from Nanjing Jiancheng Bioengineering Institute (Nanjing, China). Commercial ELISA kits for myeloperoxidase (MPO), inducible nitric oxide synthase (iNOS), tumor necrosis factor-alpha (TNF-α), interleukin-6 (IL-6), IL-17A, IL-4, IL-10, and interferon-gamma (IFN-*γ*) (Lots. 20220101-20852A, 20220101-21486A, 20220101-20852A, 20220101-20188A, 20220101-20171A, 20220101-20186A, 20220101-20162A, 20220101-20140A, respectively) were supplied by Shanghai Enzymes Biotechnology Co., Ltd. (Shanghai, China).

### 2.3 Extraction and fractionation of PA components

Dried PA powder (15 g) was extracted with ultrapure water (1:10, w/v) at 90 °C for 2 h. This extraction procedure was repeated twice. The combined aqueous extracts were filtered, and the filtrate was concentrated under reduced pressure using a rotary evaporator to a final concentration of 1.0 g/mL (equivalent to crude extract). Subsequently, anhydrous ethanol was added to the concentrate and the mixture was stored at 4 °C for 24 h to facilitate precipitation. Following centrifugation (4,000 × g, 20 min, 4 °C), the supernatant (PAW) was collected. This supernatant was sequentially fractionated by ultrafiltration using membranes with nominal molecular weight cut-offs of 10,000 Da and 3,000 Da. The resulting fractions were collected as follows: PAW1, Filtrate < 3 kDa; PAW2, Retentate 3–10 kDa (filtrate from 10 kDa membrane retained by 3 kDa membrane); PAW3, Retentate > 10 kDa. The crude PAW and the three ultrafiltered fractions (PAW1, PAW2, PAW3) were lyophilized (Freeze-dried) and stored at −20 °C until further use.

### 2.4 Nano LC-MS/MS resolution of peptide sequences in PA

The freeze-dried PA was redissolved in ultrapure water and filtered to obtain the PA test solution. The PA test solution was reduced with 10 mM of DL-dithiothreitol (DTT) at 56 °C for 1 h, followed by alkylation with 50 mM of iodoacetamide (IAA) for 40 min in the dark at room temperature to obtain the PA peptide substance. Before LC-MS/MS analysis, PA peptides were resuspended in 0.1% FA. The analysis was performed using an Ultimate 3000 System Nanoflow UPLC system (Thermo Fisher Scientific, United States) coupled to a Q Exactive™ Hybrid Quadrupole-Orbitrap™ Mass Spectrometer with an ESI nanospray source (Thermo Fisher Scientific, United States), equipped with ReproSil-Pur C18-AQ resin (150 μm × 15 cm, 1.9 μm, Dr. Maisch GmbH, Germany). Elution was performed using solvent A (2% CAN with 0.1% FA) and solvent B (80% CAN with 0.1% FA) at a 600 nL/min flow rate and an injection volume of 5 μL. The gradient elution procedure was as follows: B from 4% to 8%, 2 min; B from 8% to 28%, 43 min; B from 28% to 40%, 10 min; B from 40% to 95%, 1 min; and B from 95% to 95%, 10 min. Additionally, the samples were analyzed using a Q Exactive™ hybrid quadrupole-orbitrap™ mass spectrometer (Thermo Fisher Scientific, United States) with a spray voltage of 2.2 KV and a capillary temperature of 270 °C (Wang et al., 2023).

The mass spectrometry parameters were configured to a mass resolution of 70,000 at 400 m/z, an automatic gain control target of 3e6, a maximum fill time of 40 ms, and a mass spectrometry precursor scanning range of 300.0–1,800.0 m/z. The 20 most intense peptide ions were scanned using MS/MS starting at 100 m/z with an isolation width of 3.00 and a normalized collision energy of 40.0. Raw MS files were analyzed using Byonic and searched in the target protein database according to the sample type (Cadang et al., 2023). The parameters were set as follows: carbamoylmethylation (C) was the fixed protein modification, oxidation (M) was the variable, enzyme specificity was set to non-specific, the maximum number of missed cuts was set to 3, the precursor ion mass tolerance was set to 20 ppm, and the MS/MS tolerance was 0.02 Da (Carnevale Neto et al., 2022).

### 2.5 LC-MS resolution of molecule compounds in PA

The freeze-dried PA were dissolved in ultrapure water and filtered through a 0.22 μm microporous membrane. The target compounds were separated using an EXION LC System (SCIEX) ultra-high performance liquid chromatograph (Sciex, ExionLC AD) and passed through a Waters UPLC liquid chromatography column (ACQUITY UPLC HSS T3, 1.8 μm, 2.1 × 100 mm) at 400 μL/min (Fu et al., 2024). The mobile phases were 0.1% FA aqueous solution (A) and CAN (B), the column oven temperature was 40 °C, the autosampler temperature was 4 °C, and the injection volume was 2 μL. The gradient elution procedure was as follows: 2% B in 0–0.5 min, 2%–50% B in 0.5–10 min, 50%–95% B in 10–11 min, 95% B in 11–13 min, 95%–2% B in 13–13.1 min, and 2% B in 13.1–15 min. A SCIEX 6500 QTRAP+ triple quadrupole mass spectrometer (Sciex, Qtrap 6500 +) with an IonDrive Turbo V ESI ion source was used to perform mass spectrometry analysis in multiple reaction monitoring mode (You et al., 2024). The specific ion source parameters were as follows: ion spray voltage of +5,500/−4,500 V, curtain gas of 35 psi, temperature of 400 °C, ion source gas of 1:60 psi, ion source gas of 2:60 psi, and DP of ± 100 V (Dong et al., 2023).

### 2.6 Animal model establishment and treatment

**FIGURE 1:**
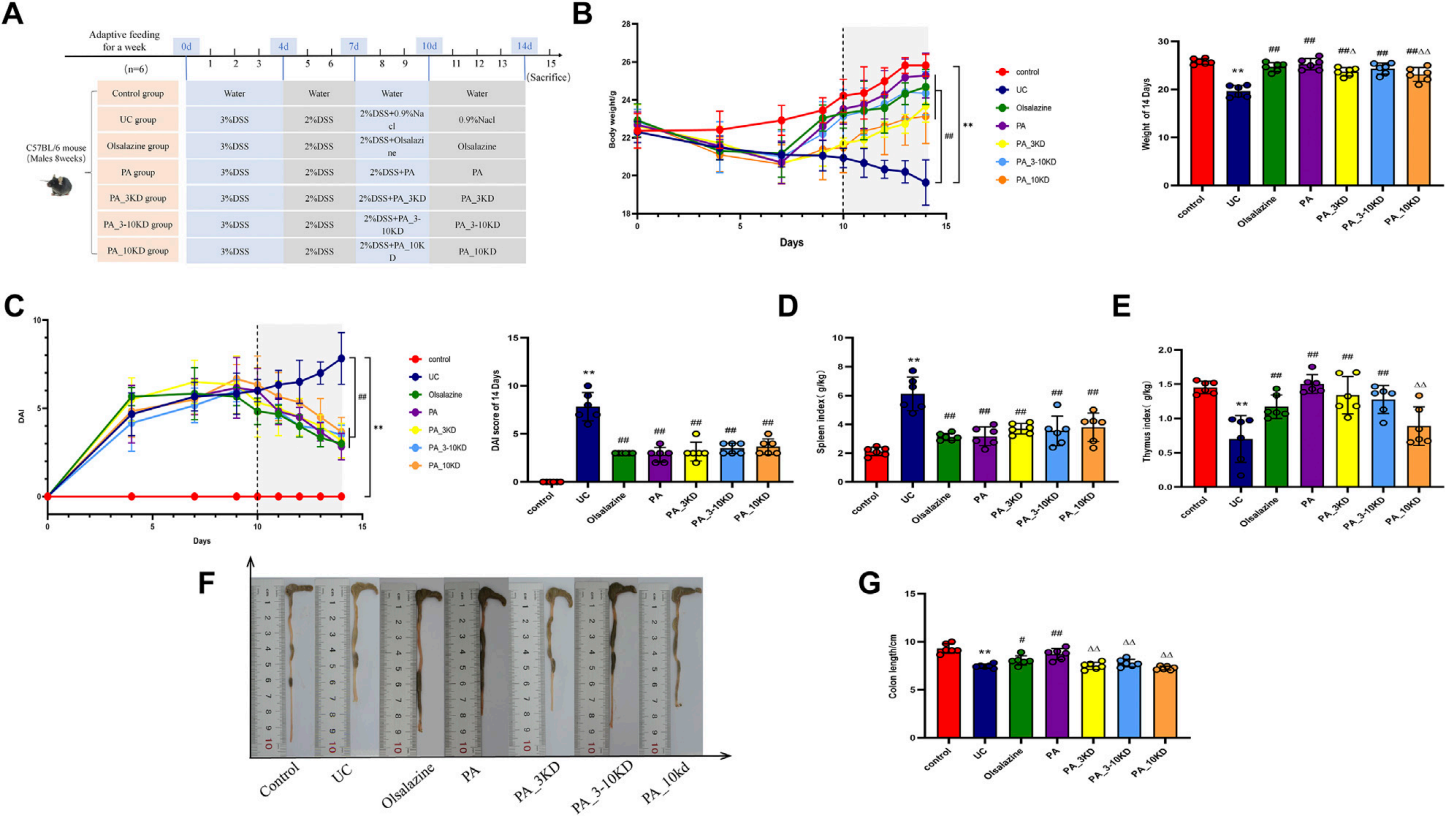
Protective effect of PA on DSS-induced UC mice. **(A)** Experimental flow design. **(B)** Changes in body weight. **(C)** Disease activity index. **(D,E)** Spleen/Thymus index. **(F,G)** Colon length. The results are expressed as the means ± SEM. ^*^ *P* < 0.05, ^**^ *P* < 0.01 vs. Control group; ^#^ *P* < 0.05, ^##^ *P* < 0.01 vs. UC group; ^Δ^ *P* < 0.05, ^ΔΔ^ *P* < 0.01 vs. PA group.

The mice were randomly divided into seven groups (n = 6 per group): control, UC model, Olsalazine (200 mg/kg), PA (100 mg/kg), PA_3KD (100 mg/kg), PA_3-10KD (100 mg/kg), and PA_10KD (100 mg/kg) group. The dosages administered were selected based on prior research findings and have been proven effective in the UC model (Tao et al., 2020; Zhang et al., 2022). Following a 1-week acclimatization period, UC was induced in all groups except the control group by administration of dextran sulfate sodium (DSS) in drinking water. Specifically, a 3% DSS solution was administered for four consecutive days, followed by a 2% DSS solution for six consecutive days (Liu et al., 2023; Zhao et al., 2024). Commencing on the eighth day of modeling, therapeutic intervention was administered for 7 days. During this phase, the control and UC model groups were treated with enemas of 0.9% NaCl solution, while the remaining groups were administered enemas containing their respective suspensions. The comprehensive experimental procedure is depicted in Figure 1A.

### 2.7 Disease activity index scoring and sample collection

Daily observations of body weight, fecal consistency, and rectal bleeding were recorded throughout the experiment. The DAI score was assigned to UC mice based on a composite of three scores: body weight loss (0, <1%; 1, 1%–5%; 2, 5%–10%; 3, 10%–20%; and 4, >20%), fecal consistency (0, normal feces; 1, well-formed pellets; 2, paste and semi-formed stools that do not adhere to the anus; 4, diarrhea, liquid stools that adhere to the anus), and rectal bleeding (0, bloodless; 1, red blood; 2, dark red blood; 4, rectal prolapse) (Niu et al., 2013). Following the completion of the treatment, the mice were humanely euthanized through cervical dislocation. Fecal samples were collected in sterile bottles on a clean bench and stored at −80 °C. The intestinal, thymus, and spleen tissues were promptly collected. The colon tissues were washed with ice saline, photographed, measured, and divided into two portions; one portion was fixed in a 4% paraformaldehyde solution, and the other was placed in an enzyme-free cryopreservation tube and stored at −80 °C. The weights of the thymus and spleen tissues were recorded. The thymus index was calculated by dividing the weight of the thymus by the weight of the body, while the spleen index was calculated by dividing the weight of the spleen by the weight of the body, expressed in grams per kilogram (Huang et al., 2024).

### 2.8 Determination of colonic cytokines and MPO and INOS activities by ELISA

The homogenized colon tissues were centrifuged at 8,000 rpm for 20 min at 4 °C, and the supernatants were analyzed for TNF-α, IL-6, IL-17A, INF-*γ*, IL-4, IL-10, INOS, and MPO using an ELISA kit according to the instructions of the manufacturers.

### 2.9 Histopathological analysis of the colon

The colon of the end tissue was fixed in 4% paraformaldehyde for 24 h, embedded in paraffin, and sectioned into 4-μm-thick slices (Nie et al., 2022). These sections were stained with hematoxylin-eosin (H&E) and alcian blue/periodic acid Schiff (AB-PAS) and examined under light microscopy to evaluate the secretory function of colonic mucus in mice and observe morphological alterations in histological sections. Histological scores (HS) were determined based on the degree of histopathologic alterations observed in colon tissue stained with H&E, which included epithelial cell changes assessment (0, normal morphology; 1, absence of cupped cells; 2, extensive loss of cupped cells; 3, deletion of crypt cells; 4, significant absence or polypoid regeneration of the crypt fossa) and cellular inflammatory infiltrates (0, none; 1, infiltration around crypts; 2, infiltration extending into the muscular layer of the mucosa; 3, widespread infiltration of the muscular layer of the mucosa with mucosal swelling; 4, infiltration into the submucosa). The overall HS was calculated by adding the scores for epithelial cell presence and cellular inflammatory infiltrate.

### 2.10 Immunohistochemistry (IHC) analysis

Samples were immersed in a sodium citrate buffer solution and heated in a microwave oven for antigenic repair. Subsequently, the sections were placed in a closed solution at room temperature for 30 min before being incubated with primary antibodies (NF-κB-p65, TLR4, MyD88, occludin-1, and ZO-1) at 4 °C overnight. All sections were treated with secondary antibodies, and the color was developed using an IHC kit (DAB). Positive DAB-stained regions were quantified using ImageJ software (version 1.48) according to the methodology reported for previous IHC analysis (Zhao et al., 2024).

### 2.11 Western blotting analysis

Total proteins were extracted from murine colon tissues utilizing a tissue protein extraction kit (Lot. 061623231130, Beyotime, China). Protein concentrations were determined using the bicinchoninic acid (BCA) protein assay kit (Lot. B500, LABLEAD, China). The extracted protein samples were denatured by boiling in sample buffer at 100 °C for 10 min and subsequently resolved via electrophoresis on a 10% sodium dodecyl sulfate-polyacrylamide gel. Proteins were then transferred onto a polyvinylidene fluoride (PVDF) membrane (Lot. ISEQ00010, Millipore, United States). Subsequently, the membranes were blocked with 5% milk for 2 h at room temperature and then incubated overnight at 4 °C with primary antibodies targeting NF-κB p65 (1:1,000, Lot 8242S, Cell Signaling Technology, United States), β-actin (1:20,000, Lot AC026, ABclonal, China), and Lamin-B (1:500, Lot WL01775, Wanleibio, China). Following incubation, the membranes underwent three washes with TBST, each lasting approximately 15 min. Thereafter, the membranes were incubated with a horseradish peroxidase (HRP)-conjugated secondary antibody (1:1,000, Lot AS014, ABclonal, China) at room temperature for 1 h. Protein expression was subsequently detected using an enhanced chemiluminescence system (Advansta, United States), and imaging was conducted with an Azure 280 imaging system (Azure Biosystems, United States). Anti-β-actin served as an internal loading control. Analysis was carried out in ImageJ and signal intensities were normalized to loading controls, where applicable.

### 2.12 16S rRNA gene sequencing

Fecal samples stored at −80 °C were removed from the refrigerator and thawed at 4 °C. Genomic DNA was extracted from feces using the aMG-Soil kit (Omega BioTek) according to the instructions of the manufacturers (Wu et al., 2022b). A 1% agarose gel was used to test the integrity of the extracted DNA, and a NanoDrop 2000 (Thermo Scientific, United States) was used to determine the concentration and purity (Li et al., 2023b). The highly variable region V3-4 of the 16S rRNA gene was PCR-amplified using an ABI GeneSide ^®^ 9700 PCR (ABI, United States) thermal cycler with the forward primer 338 F (5′- ACT​CCT​ACG​GGA​GGC​AGC​AG-3′) and reverse primer 806R (5′- GGACTACHVGGGTWTCTAAT-3′). The PCR products from the same samples were purified using 2% agarose gel electrophoresis to determine the size of the band fragments and recovered with the AxyPrepDNA Gel Kit (AXYGEN, United States). Subsequently, the purified PCR products were prepared with the NEXTFLEX Rapid DNA-Seq Kit (Bioo Scientific, Austin, United States) and sequenced on the Illumina MiSeq PE 300 platform (Illumina, San Diego, United States) following the standard protocol of Majorbio Bio-Pharm Technology Co., Ltd. The raw FASTQ files were multiplexed and quality filtered using QIIME software and subsequently aligned with high-quality 16S rRNA sequences sourced from the GreenGenes (http://greengenes.secondgenome.com/) database. The UPARSE (version 11) algorithm was employed to generate operational taxonomic units (OTUs) with a 97% similarity threshold and identify and remove chimeric sequences using UCHIME. The alpha diversity indices of the samples were assessed using Mothur software (version 1.30.2), and group differences were tested with Wilcoxon rank-sum or Kruskal–Wallis tests (with *post hoc* Dunn test). The QIIME software was utilized to conduct principal coordinate analysis (PCoA) and non-metric multidimensional scaling (NMDS) based on the Bray-Curtis distance metric to examine the resemblance of microbial community structures across the samples. Differences in relative abundances of flora at the phylum, family and genus levels were tested using Wilcoxon rank-sum and Kruskal–Wallis tests (with *post hoc* Dunn test). The LEfSe analysis software (version 1.2.11) (LDA > 2, *P* < 0.05) was used to identify bacterial taxa with significant differences in abundance from phylum to genus level among different groups. The functional gene composition of the samples was inferred using PICRUST2 software (version 2.2.0) to analyze different samples or groups for functional differences by comparing the species composition information obtained from 16S rRNA sequencing data (Douglas et al., 2020). Distance-based redundancy analysis (db-RDA) was used to investigate the effect of clinical indicators on the structure of the gut bacterial community.

### 2.13 Statistical analysis

All data are presented as the mean ± standard deviation (SD). Statistical analysis was performed using SPSS software (version 22.0). The normality of the data distribution was assessed using the Shapiro-Wilk test, and the homogeneity of variances was verified using Levene’s test. For comparisons among multiple groups (e.g., Control, UC, PA, PAW1, PAW2, PAW3), a one-way analysis of variance (ANOVA) was applied if the data met the assumptions of normality and homogeneity of variances, followed by Tukey’s *post hoc* test for pairwise comparisons. If the assumptions were violated, the non-parametric Kruskal–Wallis H test was used, followed by Dunn’s *post hoc* test with Benjamini–Hochberg correction for multiple comparisons. *P* < 0.05 was considered statistically significant. Graphs were generated using GraphPad Prism software (version 8.0.2).

## 3 Results

### 3.1 Analysis of the PA

The active constituents of the unsegmented PA extracted from *P. americana* L. were meticulously identified and characterized using Nano LC-MS/MS analysis. The comprehensive total ion chromatogram of these active compounds is depicted in Supplementary Figure S1, which recognizes a total of 438 distinct sequences (Supplementary Table S1). From this dataset, sequences that presented with a sequence score exceeding 200 and a mass accuracy deviation beyond ± 5.0 ppm were rigorously excluded. This filtration process culminated in the identification of the top 30 active component sequences, selected based on their peak area intensity, and these are systematically cataloged in Supplementary Table S2.

A total of 1,326 molecular compounds were identified by LC-MS, with detailed annotations provided in Supplementary Table S3. This chemically diverse profile encompassed phenolic compounds, amino acids and derivatives, flavonoids, fatty acyls, purine nucleosides, carboxylic acids and derivatives, alkaloids, aromatic compounds, sugars, coumarins, and plant-derived metabolites, among other classes. Notably, the following courses exhibited significant relative abundance: phenolics (25.45%), amino acids and derivatives (18.80%), nucleotides and derivatives (8.78%), carboxylic acids and derivatives (8.27%), and alkaloids (7.92%). The 30 compounds displaying the highest peak areas were rigorously selected, and their chromatographic data are presented in Supplementary Table S4. Additionally, Supplementary Figure S2 presents the total ion chromatograms (TIC) of PA extract constituents, visually illustrating the compound distribution profile.

### 3.2 PA attenuates symptoms of DSS-induced UC mice

Compared with the control mice, the UC mice exhibited a significant reduction in body weight and an increase in DAI scores (Figures 1B,C), which was reversed by administration of olsalazine, PA, and its molecular weight fractions (PA_3KD, PA_3-10KD, and PA_10KD). Remarkably, the group treated with the PA exhibited the most pronounced improvement. The indices of the thymus and spleen were also evaluated, with the UC group showing a significantly diminished thymus index and an elevated spleen index. In contrast, the treatment groups showed the opposite trend (Figures 1D,E), with the PA once again showing the most substantial therapeutic impact. Compared with the control group, the colon in the UC group exhibited a significantly shortened colon length, attributed to congestion and edema, which are key indicators of UC severity, as shown in Figures 1F,G. The PA and its fractionated components (<3, 3–10, and > 10 kDa) effectively mitigated DSS-induced colonic congestion and atrophy, thereby substantiating the therapeutic potential of PA in the treatment of UC. These findings strongly support the potential application of PA in the management and treatment of ulcerative colitis.

### 3.3 PA attenuates DSS-induced colonic histopathological damage in UC mice

**FIGURE 2:**
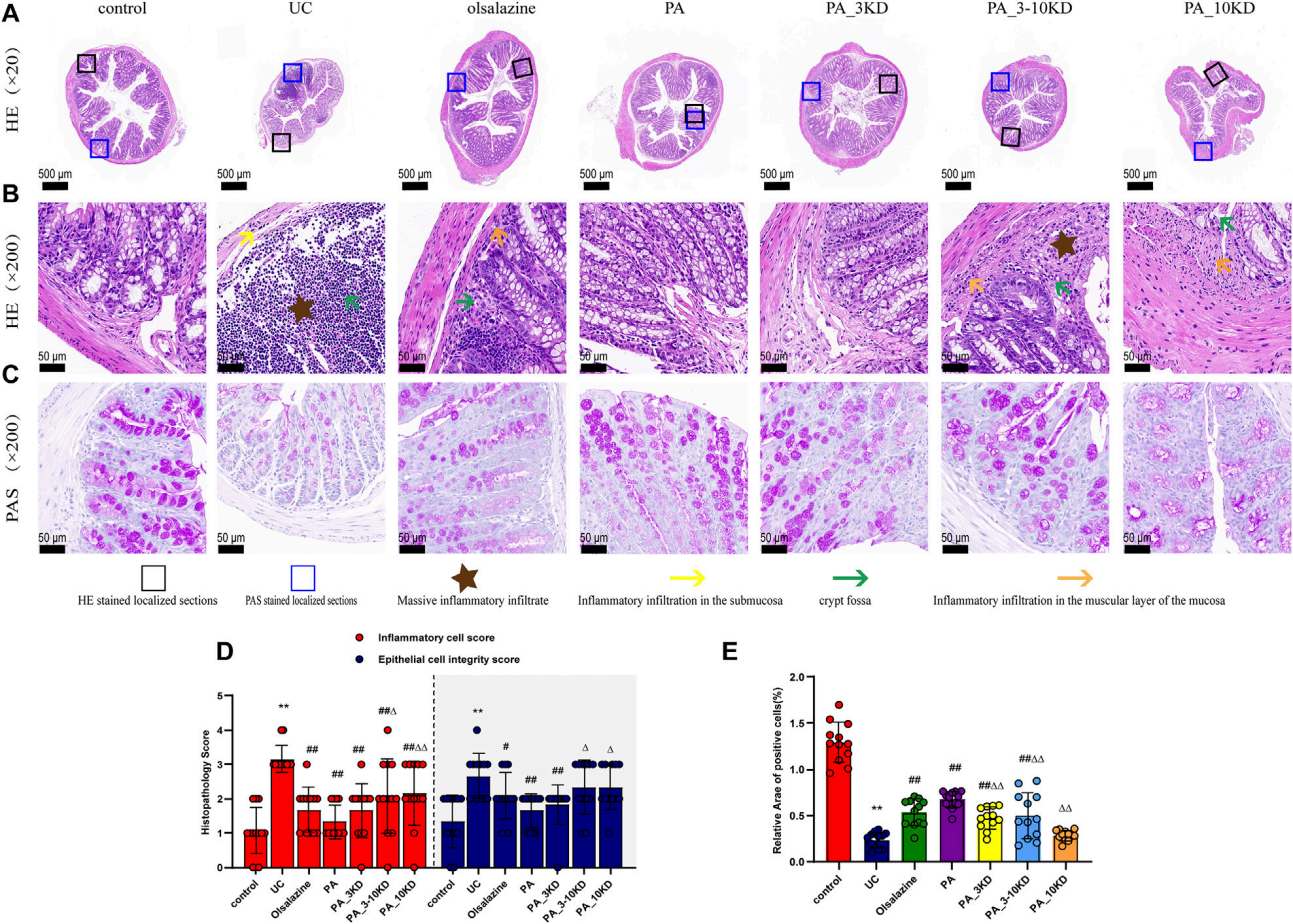
Effect of PA on pathological damage in the colon of UC mice. **(A)** The appearance of H&E-stained colon tissue (×20 magnification). **(B)** H&E staining of localized colon tissue (×200 magnification). **(C)** AB-PAS staining of localized colon tissue (×200 magnification). **(D)** Histologic scores of different groups. **(E)** Relative expression of AB-PAS-stained cup cells. The results are expressed as the means ± SEM. ^*^ *P* < 0.05, ^**^ *P* < 0.01 vs. Control group; ^#^ *P* < 0.05, ^##^ *P* < 0.01 vs. UC group; ^Δ^ *P* < 0.05, ^ΔΔ^ *P* < 0.01 vs. PA group.

H&E staining revealed the extent of colonic tissue damage and the potential protective role of PA components. In the control group, the structural integrity of the colonic tissues, including the mucosal, submucosal, and muscular layers of the mice, was intact. In contrast, mice in the UC group exhibited extensive crypt and goblet cell loss, significant inflammatory cell infiltration, and damage to the submucosal structures (Figures 2A,B). Furthermore, mice in the UC group exhibited significantly higher epithelial cell integrity and inflammatory cell scores than those in the control group. PA treatment reduced inflammatory cell infiltration, protected crypt and epithelial cell structures, and significantly decreased the colonic histology score (Figure 2D).

AB-PAS staining, which targets mucopolysaccharides and goblet cells, was an essential morphological marker for evaluating intestinal function and structure (Cheng et al., 2023a). The goblet cells of mice in the control group exhibited robust morphology and were densely arrayed flanking the crypts (Figure 2C). Conversely, those of the mice in the UC group were significantly diminished, with significantly lower relative expression rates of positive cells (Figure 2E). A significant increase in goblet cell expression was observed in the mice in the PA and PA_3KD groups than in those in the UC group following the therapeutic agent administration. This suggests that the PA components alleviated the morphological damage and restored the functional integrity of the colonic epithelium.

**FIGURE 3:**
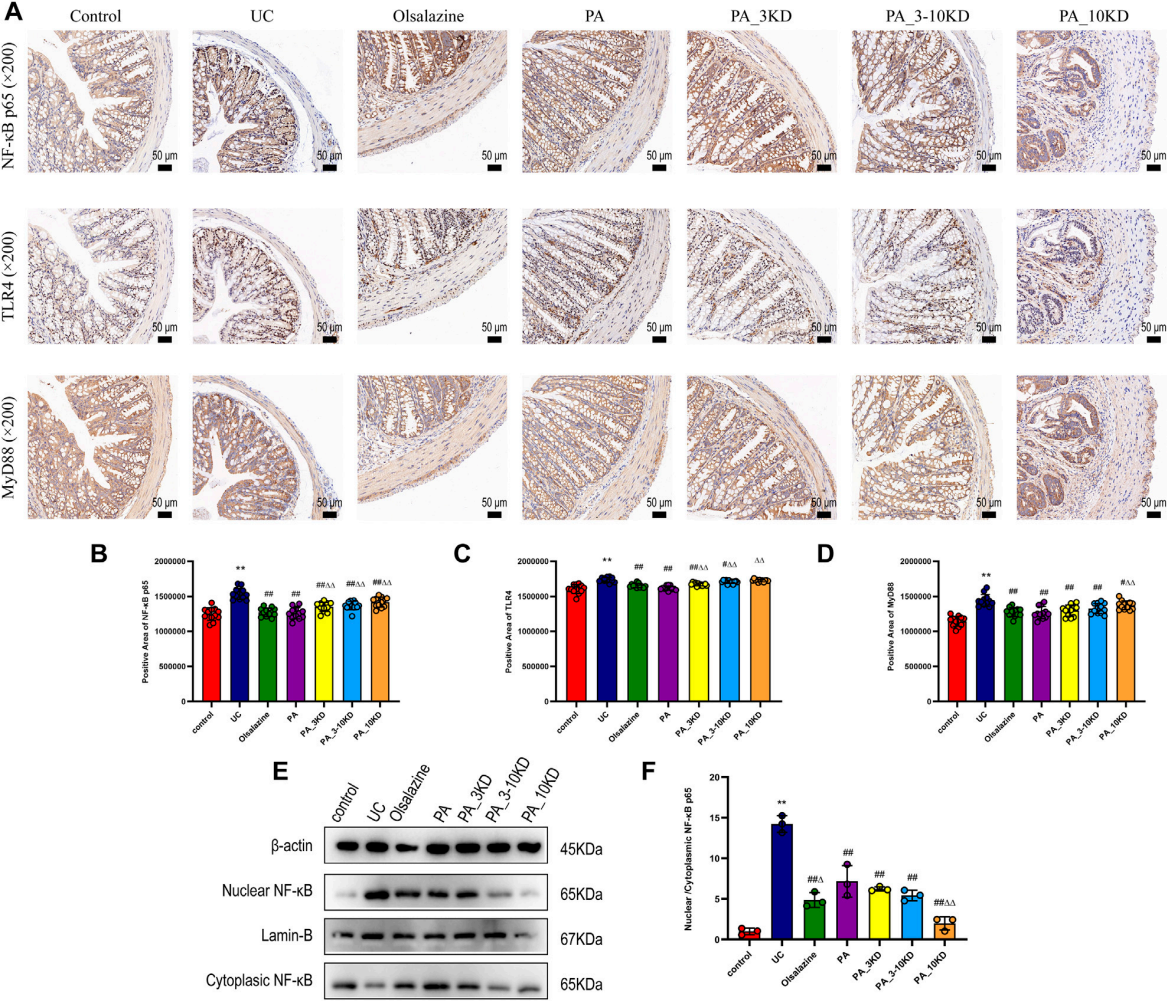
Effect of PA on inflammatory signaling at the intestinal barrier in UC mice. **(A)** IHC staining of colon NF-κB-p65, TLR4, and MyD88 of mice in each group (×200 magnification). **(B–D)** Semi-quantitative results of IHC staining analysis of NF- κB-P65, TLR4, and MyD88 proteins. The results are expressed as the means ± SEM. ^*^ *P* < 0.05, ^**^ *P* < 0.01 vs. Control group; ^#^ *P* < 0.05, ^##^ *P* < 0.01 vs. UC group; ^Δ^ *P* < 0.05, ^ΔΔ^ *P* < 0.01 vs. PA group. **(E)** The colon tissues had their cytoplasm and nucleus separated for NF-κB expression using immunoblotting analysis. **(F)** The nuclear translocation of NF-κB protein were quantified using ImageJ software.

Immunohistochemical staining revealed significant insights into the barrier function of the colonic mucosa (Figure 3). NF-κB-p65, TLR4, and MyD88 are essential in the inflammatory signaling cascade implicated in UC pathogenesis. They modulate the inflammatory response and promote pro-inflammatory cytokine synthesis, exacerbating colonic epithelial barrier dysfunction (Subramaniam et al., 2016). We observed increased NF-κB-p65, TLR4, and MyD88 expression levels among mice in the UC group, indicating an increased inflammatory response and intestinal barrier function deterioration. However, drug treatment significantly reduced the expression of these proteins in colonic tissue, with the PA blend yielding the most significant reduction (Figures 3A–D).

To investigate whether the anti-inflammatory effects of PA are associated with the NF-κB p65 signaling pathway, the expression levels of NF-κB p65 in both the nucleus and cytoplasm were assessed. As illustrated in Figures 3E,F, there was an overexpression of NF-κB p65 in the nucleus and a concomitant reduction in the cytoplasm within the colon tissues of the UC group. This observation suggests that UC facilitates the nuclear translocation of NF-κB p65, thereby activating the NF-κB p65 pathway (*P* < 0.05). Conversely, PA treatment markedly inhibited the nuclear translocation of NF-κB p65 in mice with colitis. These findings indicate that PA treatment mitigates the inflammatory response by suppressing the activation of the NF-κB p65 signaling pathway.

**FIGURE 4:**
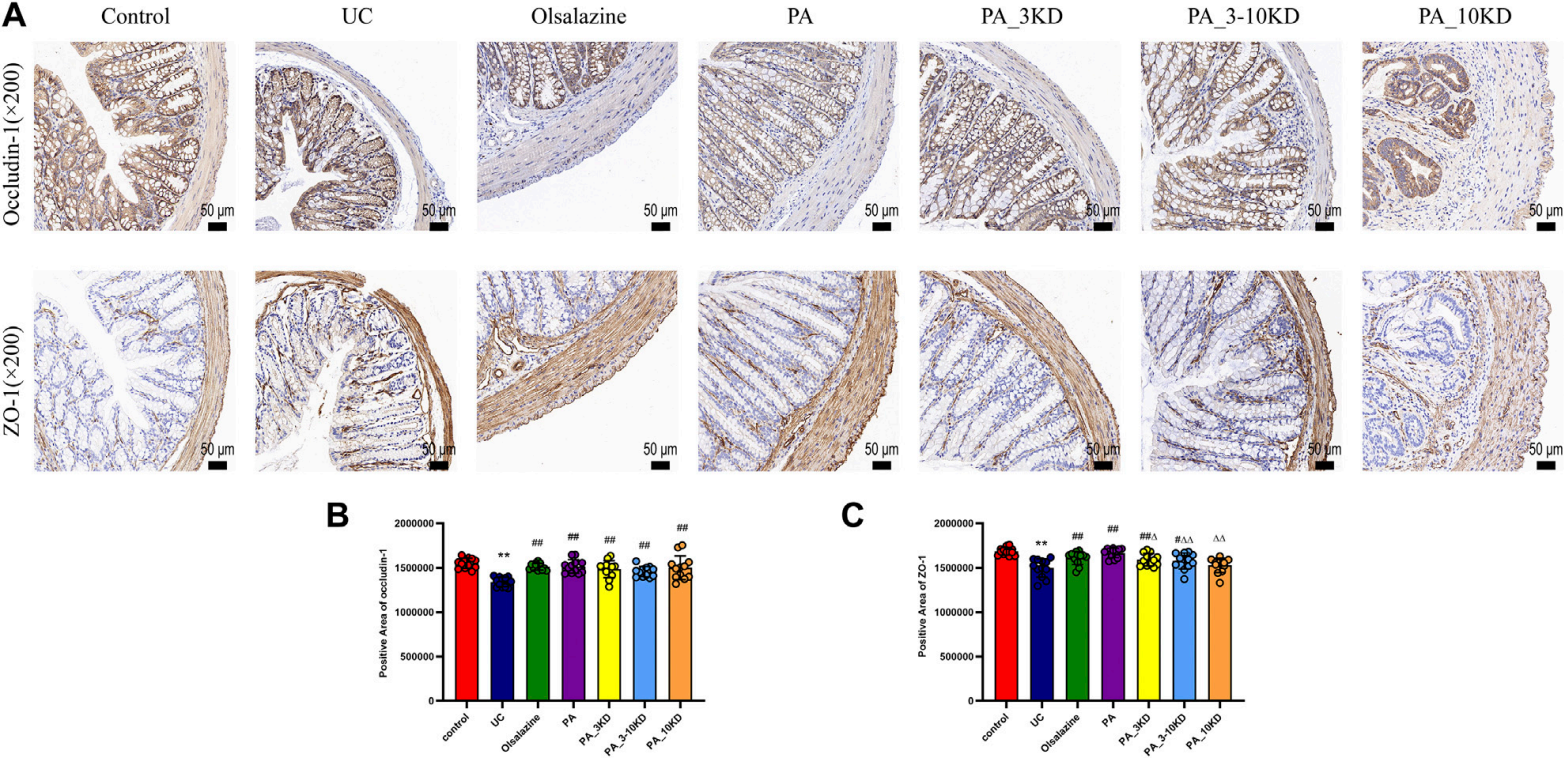
Effect of PA on intestinal barrier integrity in UC mice. **(A)** IHC staining of colon ZO-1 and occludin-1 of mice in each group (×200 magnification). **(B, C)** Semi-quantitative results of IHC staining analysis of ZO-1 and occludin-1 proteins. The results are expressed as the means ± SEM. ^*^ *P* < 0.05, ^**^ *P* < 0.01 vs. Control group; ^#^ *P* < 0.05, ^##^ *P* < 0.01 vs. UC group; ^Δ^ *P* < 0.05, ^ΔΔ^ *P* < 0.01 vs. PA group.

Occludin-1 and ZO-1 were identified as key components of the tight junctions that constitute the mechanical barrier between intestinal epithelial cells. The mice in the UC group demonstrated significantly reduced occludin-1 and ZO-1 expression levels than those in the control group, indicating a breakdown of intestinal barrier integrity (Figures 4A–C). However, PA treatment significantly increased occludin-1 and ZO-1 expression, indicating intestinal barrier restoration. The PA significantly increased occludin-1 levels. These findings revealed that PA components are efficacious in mitigating the DSS-induced intestinal barrier dysfunction in UC, highlighting their potential therapeutic value.

### 3.4 DSS-induced changes in colonic inflammatory factors in UC mice

**FIGURE 5:**
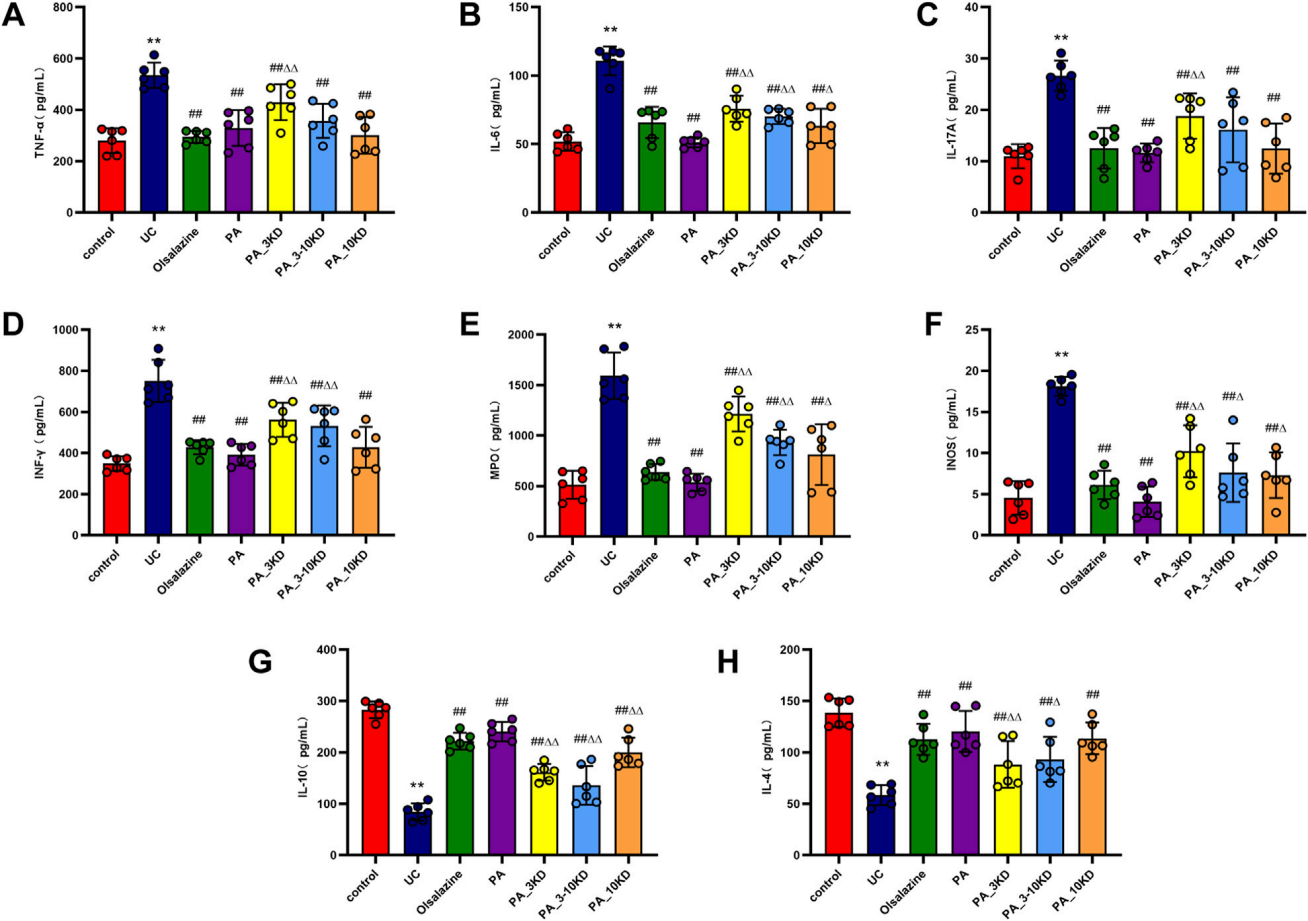
Effect of PA on colonic inflammatory factors. **(A)** TNF-α, **(B)** IL-6, **(C)** IL-17A, **(D)** INF-*γ*, **(E)** MPO, **(F)** INOS, **(G)** IL-4, **(H)** IL-10. The results are expressed as the means ± SEM. ^*^ *P* < 0.05, ^**^ *P* < 0.01 vs. Control group; ^#^ *P* < 0.05, ^##^ *P* < 0.01 vs. UC group; ^Δ^ *P* < 0.05, ^ΔΔ^ *P* < 0.01 vs. PA group.

The concentration of inflammatory cytokines within the colonic mucosa is a strong indicator of UC disease severity (Cheng et al., 2023b). Figure 5 indicates that UC-affected mice have significantly higher levels of pro-inflammatory mediators, including TNF-α, IL-6, IL-17A, and INF-*γ*, than those in the control group (Figures 5A–D). Simultaneously, increased MPO and INOS expressions were observed in the UC cohort (Figures 5E,F). Overproduction of these pro-inflammatory cytokines was considered a central factor in UC development and exacerbation. This study demonstrates that the administration of PA and its various molecular weight fractions significantly reduced the levels of these inflammatory mediators in mice in the PA groups than in those in the untreated UC group. The complete PA extract was more effective than its components in diminishing the levels of pro-inflammatory cytokines, MPO, and INOS, suggesting a synergistic effect of the combined ingredients in PA anti-inflammatory action.

In contrast to pro-inflammatory mediators, we found that post-treatment levels of anti-inflammatory cytokines (IL-4 and IL-10) were significantly higher in the mice in the UC model group than in those in the control group (Figures 5G,H). PA administration was significantly effective in boosting these anti-inflammatory cytokine levels. These findings indicate that the PA extract reduces inflammation in the UC mouse model by suppressing pro-inflammatory factors expression and increasing anti-inflammatory mediators’ expression. Accordingly, the extract may play a dual modulatory role in the inflammatory response, potentially contributing to its therapeutic efficacy in UC management.

### 3.5 Effects of PA on intestinal flora

**FIGURE 6:**
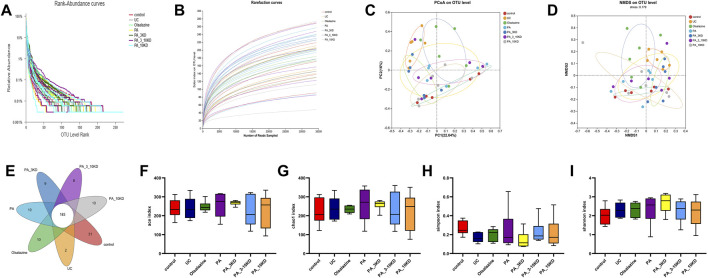
PA regulates the diversity of gut microbiota in UC mice. **(A)** The Rank-abundance plot in each group. **(B)** The Rarefaction curves on levels in each group. **(C, D)** The β-diversity analysis is based on principal coordinate analysis (PCoA) and non-metric multidimensional scaling analysis (NMDS) based on Bray-Curtis distance for all samples. **(E)** The number of unique OUT levels that are unique to each group. **(F)** Ace index. **(G)** Chao1 index. **(H)** Simpson index. **(I)** Shannon index. The results are expressed as the means ± SEM [**(A–I)** n = 6].

The utilization of 16S rRNA gene sequencing to examine the effect of PA on intestinal microflora diversity and composition in a DSS-induced UC mouse model highlights the role of the gut microbiome in UC and the potential therapeutic effects of PA. These findings demonstrated that the sequencing effort was sufficient to capture a representative snapshot of microbial richness across the samples, as indicated by the plateauing of the rarefaction curves (Figures 6A,B). Microbiome studies need this validation step to ensure that further analysis is based on a comprehensive dataset.

The PCoA and NMDS analyses (Figures 6C,D) are multivariate statistical techniques used for reducing the dimensionality of complex data sets. The significant spatial separation between mice in the UC model group and those in the control group in these analyses indicates that UC causes a distinct shift in the gut microbiota composition. The fact that the gut microbiota composition of mice in the groups treated with various molecular weight fractions of PA is closer to that of mice in the control group suggests that these treatments can alter the gut microbiota to mimic that of the healthy controls.

The identification of 263 OTUs across all samples, with 183 OTUs shared by all groups, indicates that a core microbiome exists under all conditions. The unique OTUs found in each treatment group (Figure 6E) may be of particular interest because they may represent specific bacterial taxa associated with the therapeutic effects of PA and its fractions.

The alpha diversity analysis (Figures 6F–I), utilizing the Kruskal–Wallis test, indicated no significant differences in the Ace (*H* = 2.412, *df* = 6, *P* = 0.878), Chao1 (*H* = 2.230, *df* = 6, *P* = 0.897), Shannon (*H* = 4.777, *df* = 6, *P* = 0.573), and Simpson (*H* = 8.726, *df* = 6, *P* = 0.190) indices across groups. This lack of significant variation was corroborated by Dunn’s test with Benjamini–Hochberg correction, which also revealed no significant differences between groups (all adjusted *P* > 0.05). These findings suggest that DSS-induced colitis and PA treatment did not significantly impact overall microbial abundance and homogeneity. However, the moderate effect sizes (*ε* ^2^ = 0.12–0.21) indicate that further validation with an expanded sample size is necessary to explore potential underlying biological trends. Detailed results are presented in Tables 1 and 2.

**TABLE 1:** Statistical test results for alpha diversity indices between groups.

Alpha diversity	N	*H*	*df*	*P*	*ε* ^2^
Ace	42	2.421	6	0.878	0.05904878
Chao	42	2.230	6	0.897	0.054390244
Shannon	42	4.777	6	0.573	0.116512195
simpson	42	8.726	6	0.190	0.212829268

Epsilon-squared (*ε* ^2^) is calculated as: *ε* ^2^ = *H*/[*N* (*N* + 1)/(*N* − 1)], N represents the total sample size. *ε* ^2^ < 0.08 weak effect, which may lack biological significance; *ε* ^2^ = 0.08–0.26 moderate effect, whose interpretation should be supported by additional evidence; *ε* ^2^ > 0.26 strong effect, providing support for the core hypothesis.

**TABLE 2:** Comparison of median alpha diversity indices among groups.

Group	Median				
	n	Ace	Chao 1	Shannon	Simpson
Control	6	232.24	206.14	2.03	0.25
UC	6	233.38	234.77	2.28	0.16
Olsalazine	6	243.24	234.91	2.39	0.22
PA	6	274.69	271.41	2.57	0.17
PA_3KD	6	270.55	264.13	2.79	0.11
PA_3-10KD	6	206.60	207.48	2.38	1.86
PA_10KD	6	256.98	249.02	2.30	0.17

**FIGURE 7:**
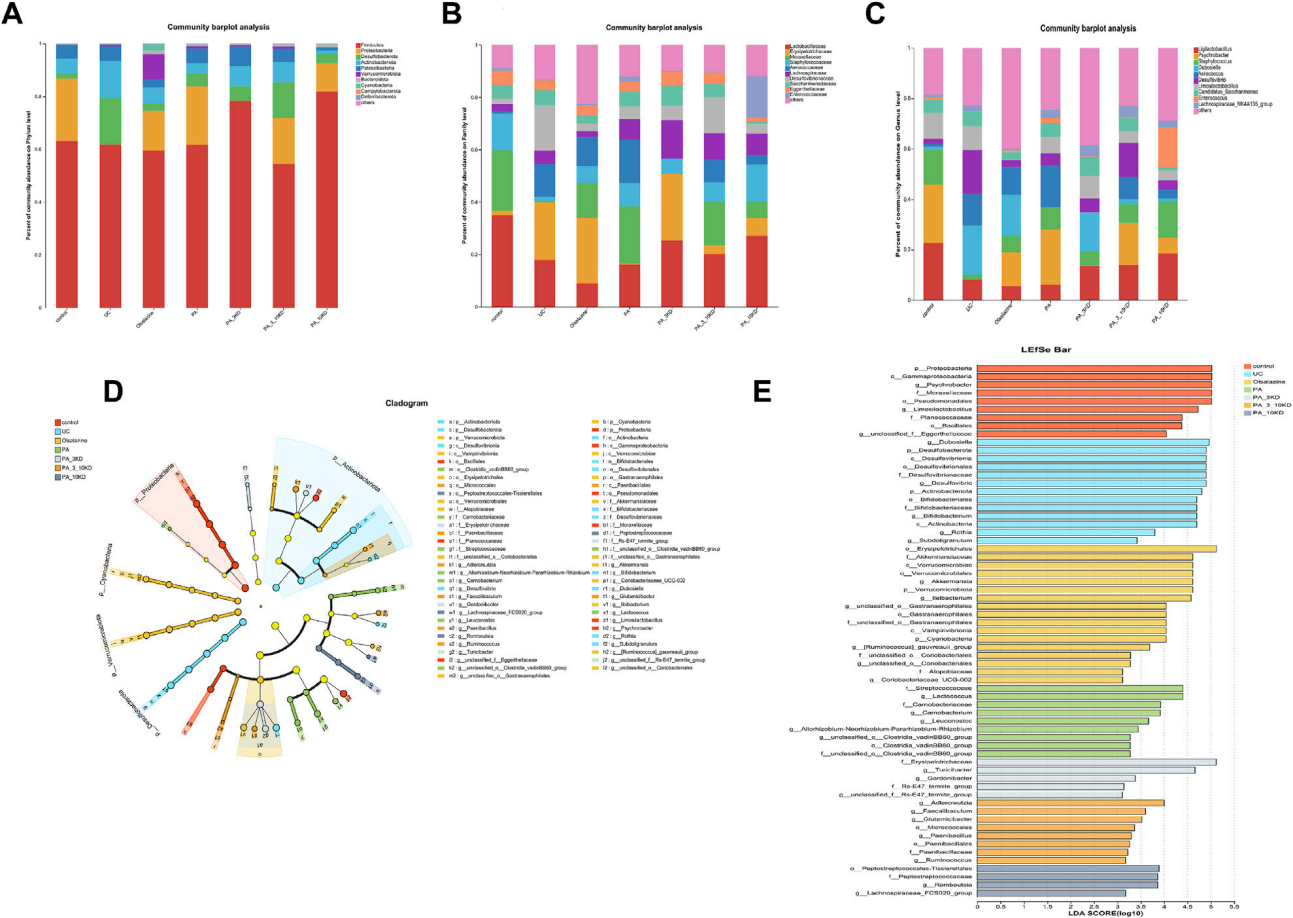
Changes in the gut microbiota. **(A–C)** Relative abundance of species at the phylum level, abundance heatmap, and differential analysis of relative abundance of the top 10 colonies at the phylum level. **(D)** Taxonomic branching diagrams obtained from LefSe analysis. **(E)** Linear discriminant analysis effect size (LefSe) analysis of the dominant biomarker taxa in each group level (LDA > 3.0).

This study identified species from 11 phyla, 17 classes, 45 orders, 78 families, 170 genera, and 654 OTUs.Through a comprehensive multilevel taxonomic analysis encompassing the phylum, family, and genus levels, distinct variations in the floral structure among the groups were identified (Figures 7A–C). At the phylum level, the Control group was predominantly composed of *Firmicutes* (63.2%) and *Proteobacteria* (23.5%). In contrast, the UC group exhibited a marked increase in *Desulfobacterota* (17.4% vs. 2.03%) and *Actinobacteriota* (14.08% vs. 5.60%), alongside a significant reduction in *Proteobacteria* (0.06% vs. 23.5%). The Kruskal–Wallis test indicated significant intergroup differences across five phyla, notably *Proteobacteria* (*H* = 16.133, *P* = 0.013, *ε* ^2^ = 0.393) and *Verrucomicrobiota* (*H* = 25.689, *P* < 0.001, *ε* ^2^ = 0.627), as presented in Table 3. Notably, the PA intervention significantly reinstated the abundance of *Proteobacteria* (compared to UC: *H* = 19.083, *Z* = 2.695, *P* = 0.007). Furthermore, both PA and PA_10KD interventions effectively mitigated the abnormal increases in *Deferribacterota* (*H* = 17.750/14.667, *Z* = 3.145/2.598, *P* ≤ 0.009) and *Desulfobacterota* (*H* = 16.667/19.333, *Z* = 2.353/2.730, *P* ≤ 0.019), as detailed in Table 4. At the family level (Table 5), the UC group exhibited a significant enrichment in *Akkermansiaceae* (*H* = 23.500, *Z* = 3.321, *P* = 0.001), *Bifidobacteriaceae* (*H* = 23.083, *Z* = 3.326, *P* = 0.001), and *Erysipelotrichaceae* (*H* = 20.917, *Z* = 2.953, *P* = 0.003), while *Moraxellaceae* (*H* = 22.500, *Z* = 3.206, *P* = 0.001) was significantly reduced. This microbial imbalance was effectively reversed by PA and PA_3-10KD treatments. At the genus level (Table 6), there was a significant elevation in *Akkermansia*, *Bifidobacterium*, *Dubosiella* and *Faecalibaculum* within the UC group (*P* < 0.01). However, PA treatment significantly reduced the abundance of *Bifidobacterium* (*H* = 26,000, *Z* = 3,746, *P* < 0.001), *Dubosiella* (*H* = 24,750, *Z* = 3,496, *P* < 0.001), and *Faecalibaculum* (*H* = 22,500, *Z* = 3,188, *P* < 0.001). These findings suggest that PA may ameliorate UC pathology by modulating specific microbial communities.

**TABLE 3:** Statistical analysis of top 5 compositional differences at phylum, family, and genus levels among groups.

Level	Name	N	*H*	*df*	*P*	*ε* ^2^
Phylum	*Actinobacteriota*	42	15.628	6	0.016	0.381170732
	*Deferribacterota*	42	14.786	6	0.022	0.360634146
	*Desulfobacterota*	42	14.35	6	0.026	0.350000000
	*Proteobacteria*	42	16.133	6	0.013	0.393487805
	*Verrucomicrobiota*	42	25.689	6	0.000	0.626560976
Family	*Akkermansiaceae*	42	25.689	6	0.000	0.626560976
	*Bifidobacteriaceae*	42	25.028	6	0.000	0.610439024
	*Erysipelotrichaceae*	42	24.818	6	0.000	0.605317073
	*Exiguobacteraceae*	42	18.900	6	0.004	0.460975610
	*Moraxellaceae*	42	17.560	6	0.007	0.428292683
Genus	*Akkermansia*	42	25.689	6	0.000	0.626560976
	*Bifidobacterium*	42	25.018	6	0.000	0.610195122
	*Dubosiella*	42	23.132	6	0.001	0.564195122
	*Faecalibaculum*	42	21.765	6	0.001	0.530853659
	*Ruminococcus*	42	25.822	6	0.000	0.629804878

**TABLE 4:** Pairwise comparisons of significantly differential taxa at the phylum level.

Phylum	Comparison	*H*	Z-score Dunn	*P*	Pcorr Dunn
*Actinobacteriota*	PA_10KD - UC	26.000	3.671	0.000	0.005
	PA_10KD - Olsalazine-	17.333	2.447	0.014	0.302
	PA_10kD - PA_3KD	20.500	2.894	0.004	0.080
*Deferribacterota*	Control - UC	−17.750	−3.145	0.002	0.035
	PA - UC	17.750	3.145	0.002	0.035
	PA_3-10KD - UC	13.500	2.392	0.017	0.352
	PA_10KD - UC	14.667	2.598	0.009	0.197
*Desulfobacterota*	Control - UC	−22.333	−3.153	0.002	0.034
	Olsalazine - UC	22.167	3.130	0.002	0.037
	PA - UC	16.667	2.353	0.019	0.391
	PA_3KD - UC	14.833	2.094	0.036	0.761
	PA_10KD - UC	19.333	2.730	0.006	0.133
*Proteobacteria*	Control - UC	−20.750	−2.931	0.003	0.071
	Control - PA_3KD	−18.083	−2.554	0.011	0.224
	Olsalazine - UC	18.583	2.625	0.009	0.182
	PA - UC	19.083	2.695	0.007	0.148
	PA_3-10KD - UC	14.083	1.989	0.047	0.98
	PA_3KD - Olsalazine	15.917	2.248	0.025	0.516
	PA_3KD - PA	16.417	2.319	0.020	0.429
*Verrucomicrobiota*	Control - UC	−23.500	−3.321	0.001	0.019
	Control - Olsalazine	−29.417	−4.157	0.000	0.001
	Control - PA	−15.667	−2.214	0.027	0.563
	Control - PA_3KD	−20.333	−2.873	0.004	0.085
	Control- PA_3-10KD	−21.417	−3.027	0.002	0.052
	PA_10KD - UC	17.750	2.508	0.012	0.255
	PA_10KD - Olsalazine	23.667	3.345	0.001	0.017
	PA_10KD - PA_3KD	14.583	2.061	0.039	0.826
	PA_10KD - PA_3-10KD	15.667	2.214	0.027	0.563

**TABLE 5:** Pairwise comparisons of significantly differential taxa at the family level.

Family	Comparison	*H*	Z-score Dunn	*P*	Pcorr Dunn
*Akkermansiaceae*	Control - UC	−23.500	−3.321	0.001	0.019
	Control - Olsalazine	−29.417	4.157	0.000	0.001
	Control - PA	−15.667	2.214	0.027	0.563
	Control - PA_3KD	−20.333	2.873	0.004	0.085
	Control - PA_3-10KD	−21.417	3.027	0.002	0.052
	PA_10KD - UC	17.750	2.508	0.012	0.255
	PA_10KD - Olsalazine	23.667	3.345	0.001	0.017
	PA_10KD - PA_3KD	14.583	2.061	0.039	0.826
	PA_3-10KD - PA_3KD	15.667	2.214	0.027	0.563
*Bifidobacteriaceae*	Control - UC	−23.083	3.326	0.001	0.019
	Control - Olsalazine	−17.083	2.461	0.014	0.291
	PA - UC	26.000	3.746	0.000	0.004
	PA_3-10KD - UC	19.583	2.822	0.005	0.100
	PA_10KD - UC	22.917	3.302	0.001	0.020
	PA - Olsalazine	20.000	2.882	0.004	0.083
	PA_10KD - Olsalazine	16.917	2.437	0.015	0.311
	PA_3KD - PA	16.250	2.341	0.019	0.404
*Erysipelotrichaceae*	Control - UC	−20.917	2.953	0.003	0.066
	Control - Olsalazine	−16.583	2.341	0.019	0.403
	Control - PA_3KD	−18.583	2.624	0.009	0.183
	PA - UC	25.417	3.589	0.000	0.007
	PA_3-10KD - UC	19.833	2.800	0.005	0.107
	PA_10KD - UC	14.667	2.071	0.038	0.806
	PA - Olsalazine	21.083	2.977	0.003	0.061
	PA_3-10KD - Olsalazine	15.500	2.188	0.029	0.601
	PA_3KD - PA	23.083	3.259	0.001	0.023
	PA_3-10KD - PA_3KD	17.500	2.471	0.013	0.283
*Exiguobacteraceae*	Control - PA_3-10KD	−10.500	3.320	0.001	0.019
	PA_3-10KD - UC	10.500	3.320	0.001	0.019
	PA_3-10KD - Olsalazine	10.500	3.320	0.001	0.019
	PA_3-10KD - PA	10.500	3.320	0.001	0.019
	PA_3-10KD - PA_3KD	10.500	3.320	0.001	0.019
	PA_3-10KD - PA_10KD	10.500	3.320	0.001	0.019
*Moraxellaceae*	Control - UC	−22.500	3.206	0.001	0.028
	Control - PA_3KD	−17.333	2.470	0.014	0.284
	Control - PA_10KD	−14.500	2.066	0.039	0.815
	Olsalazine - UC	16.833	2.399	0.016	0.345
	PA - UC	20.667	2.945	0.003	0.068
	PA_3-10KD - UC	16.667	2.375	0.018	0.368
	PA_3KD - PA	15.500	2.209	0.027	0.571

**TABLE 6:** Pairwise comparisons of significantly differential taxa at the genus level.

Genus	Comparison	*H*	Z-score Dunn	*P*	Pcorr Dunn
*Akkermansia*	Control - UC	−23.500	−3.321	0.001	0.019
	Control - Olsalazine	−29.417	−4.157	0.000	0.001
	Control - PA	−15.667	−2.214	0.027	0.563
	Control - PA_3KD	−20.333	−2.873	0.004	0.085
	Control - PA_3-10KD	−21.417	−3.027	0.002	0.052
	PA_10KD - UC	17.750	2.508	0.012	0.255
	PA_10KD - Olsalazine	23.667	3.345	0.001	0.017
	PA_10KD - PA_3KD	14.583	2.061	0.039	0.826
	PA_10KD - PA_3-10KD	15.667	2.214	0.027	0.563
*Bifidobacterium*	Control - UC	−23.083	−3.326	0.001	0.019
	Control - Olsalazine	−17.083	−2.461	0.014	0.291
	PA - UC	26.000	3.746	0.000	0.004
	PA_3-10KD - UC	19.583	2.822	0.005	0.100
	PA_10KD - UC	22.917	3.302	0.001	0.020
	PA - Olsalazine	20.000	2.882	0.004	0.083
	PA_10KD - Olsalazine	16.917	2.437	0.015	0.311
	PA_3KD - PA	16.250	2.341	0.019	0.404
*Dubosiella*	Control - UC	−17.667	−2.496	0.013	0.264
	Control - Olsalazine	−14.000	−1.978	0.048	1.000
	PA - UC	24.750	3.496	0.000	0.010
	PA_3-10KD - UC	17.000	2.401	0.016	0.343
	PA_10KD - UC	23.917	3.378	0.001	0.015
	PA - Olsalazine	21.083	2.978	0.003	0.061
	PA_10KD - Olsalazine	20.250	2.861	0.004	0.089
	PA_3KD - PA	17.250	2.437	0.015	0.311
	PA_10KD - PA_3KD	16.417	2.319	0.020	0.428
*Faecalibaculum*	Control - UC	−14.417	−2.043	0.041	0.863
	Control - Olsalazine	−17.417	−2.468	0.014	0.286
	PA - UC	22.500	3.188	0.001	0.030
	PA_10KD - UC	15.500	2.196	0.028	0.590
	PA - Olsalazine	25.500	3.613	0.000	0.006
	PA_10KD - Olsalazine	18.500	2.621	0.009	0.184
	PA_3KD - PA	21.417	3.034	0.002	0.051
	PA_3-10KD - PA	15.833	2.243	0.025	0.522
	PA_10KD - PA_3KD	14.417	2.043	0.041	0.863
*Ruminococcus*	Control - Olsalazine	−14.000	−3.881	0.000	0.002
	UC - Olsalazine	−14.000	−3.881	0.000	0.002
	PA - Olsalazine	14.000	3.881	0.000	0.002
	PA_3KD - Olsalazine	14.000	3.881	0.000	0.002
	PA_3-10KD - Olsalazine	14.000	3.881	0.000	0.002
	PA_10KD - Olsalazine	14.000	3.881	0.000	0.002

A comprehensive analysis was performed on all samples at the phylum to genus level, utilizing the LefSe method with an LDA threshold of >3.0 to distinguish distinct bacterial communities under varying conditions. The results were presented using branching plots and histograms to illustrate the distribution of LDA values (Figures 7D,E). Branching plots revealed significant differences in gut microbial taxa between groups, while histograms of LDA scores revealed the degree of influence of significantly different species, resulting in the identification of 62 microbial markers. The number of microorganisms that differed between the control, UC, oxalazine, PA, PA_3KD, PA_3-10KD, and PA_10KD groups were 8, 12, 12, 11, 6, 9, and 4. Among them, four key genera, *Psychrobacter*, *Limosilactobacillus*, *unclassified*, and *Jeotgalicoccus,* were found in the mice in the control group, while those found in the mice in the UC group included *Dubosiella*, *Desulfovibrionia*, *Bifidobacterium*, and *Clostridium sensu stricto 1*.

### 3.6 Relevance analysis and functional prediction

**FIGURE 8:**
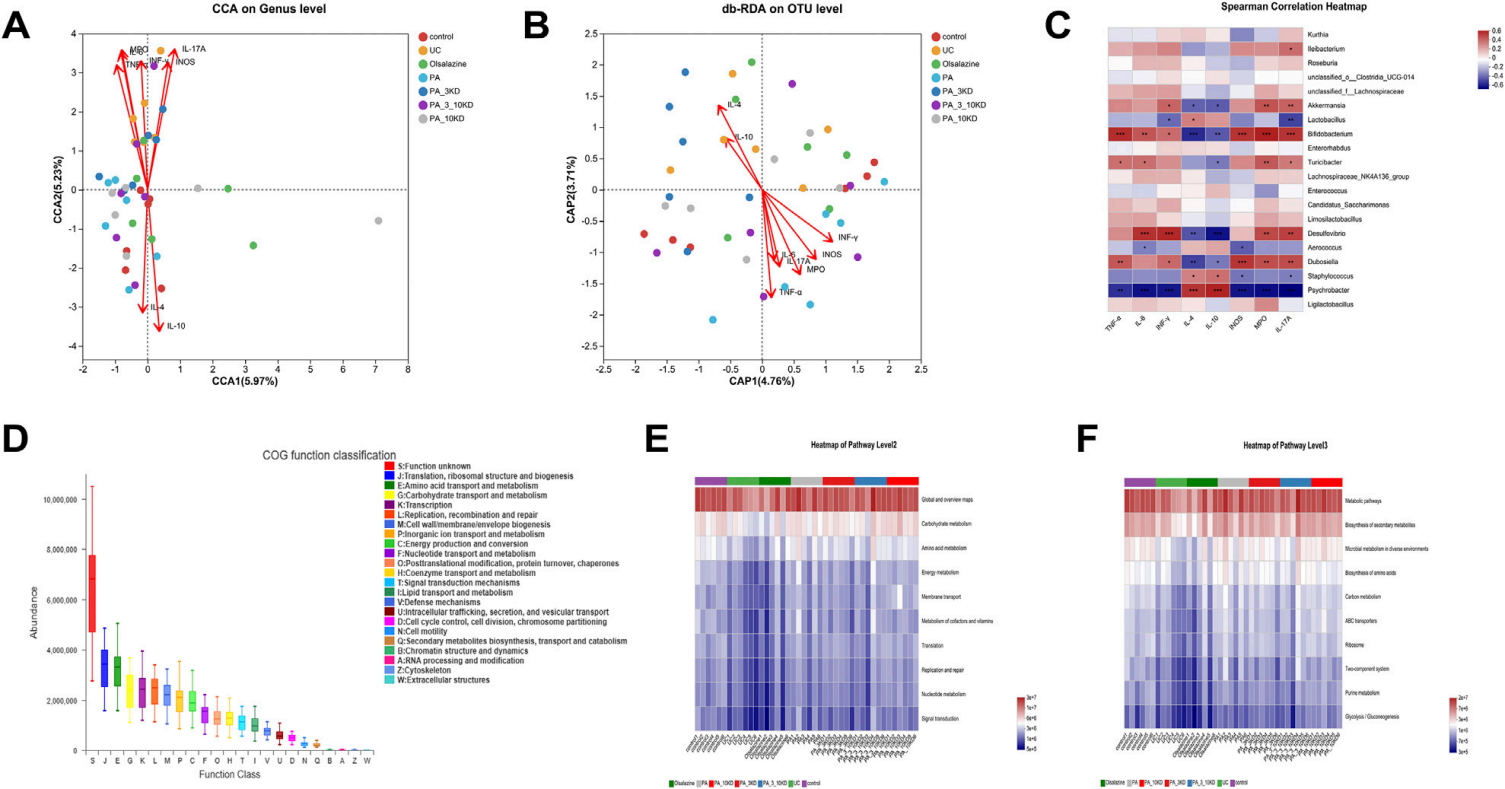
Correlation analysis and functional prediction of gut microbiota with inflammatory factors. **(A)** RDA/CAA environmental factor analysis shows the extent to which inflammatory factors influence microbiota composition. **(B,C)** Db-RDA analysis and Spearman’s correlation heat map to analyze the correlation between inflammatory factors and gut microbiota. **(D)** PICRUSt2 combined with the egNOG database to predict the function of bacterial microbiota in tissues. **(E,F)** PICRUSt2 combined with the KEGG database predicts bacterial microbial communities in tissues as a function of the KEGG pathway at level 2 and level 3.

The above findings suggest that PA effectively reduces intestinal inflammation and modulates intestinal flora composition in UC mice. However, the potential impact of PA on the relationship or coherence between these two factors remains unclear. The effects of cytokines on gut microbiota composition were assessed using canonical correlation analysis (RDA/CCA), db-RDA, and Spearman correlation heatmaps (Figures 8A–C). In RDA/CCA and db-RDA analyses microbes in the gut exhibited significant positive correlations with anti-inflammatory factors (IL-4 and IL-10) and negative correlations with pro-inflammatory cytokines (TNF-α, IL-6, IL-17A, INF-*γ*, INOS, and MPO). Among them, IL-10, IL-17A, and MPO exhibited the most significant influence on the changes in intestinal flora in UC mice. The Spearman correlation heatmaps depicted in Figure 8C indicate a positive correlation between *Psychrobacter* and anti-inflammatory cytokines (IL-4 and IL-10), suggesting that *Psychrobacter* may play a role in the regulation of inflammation and intestinal flora composition. Conversely, *Dubosiella, Bifidobacterium, Akkermansia, and Desulfovibrio* exhibited contrasting associations. These findings imply that modulating the abundance and composition of *Psychrobacter, Dubosiella, Bifidobacterium, Akkermansia, and Desulfovibrio* flora in UC mice may provide a therapeutic approach to alleviate DSS-induced intestinal inflammation.

The functions of microbial communities were predicted using PICRUSt2 software combined with EggNOG (evolutionary genealogy of genes: non-supervised orthologous groups) and Kyoto encyclopedia of genes and genomes (KEGG) databases based on the 16S rRNA amplicon sequencing results (Figures 8D–F). Microorganisms predicted from the eggNOG database have known functions primarily related to biosynthesis and metabolism, with translation, ribosomal structure, and biogenesis accounting for the highest abundance. Similarly, KEGG function predictions indicate that microbial community function is primarily associated with carbohydrate, amino acid, energy, and nucleotide metabolic pathways. When combined with the informative expression of KEGG data at a deeper (tertiary) level, the microbial association with secondary metabolites and amino acid biosynthesis, carbon metabolism, purine metabolism, and glycolysis was confirmed.

## 4 Discussion

*P. americana* L. is a traditional Chinese medicine with a long history of medicinal use, and modern pharmacological studies have confirmed that its extracts have a variety of biological activities, such as anti-inflammatory, antimicrobial, tissue repair, and immunomodulation (Peng et al., 2024; Zhao et al., 2017). Its active ingredients have been developed into a variety of modern preparations (Kangfuxin solut, Xinmailong injection, and Ganlong capsule) for clinical application. Our previous study found that its alcoholic extract (Ento-A) could repair the colonic mucosal barrier by regulating the intestinal flora and had therapeutic effects on ulcerative colitis (UC). However, whether its aqueous extract (PA) has the same therapeutic effect, the active substance basis, and the mechanism of action for the treatment of UC are not clear. Therefore, we proposed an analysis of the peptide and molecular components of PA using mass spectrometry, categorizing them by molecular weight (<3, 3–10, >10 kDa). This was integrated with a DSS-induced UC mouse model to comprehensively evaluate the therapeutic effects of PA compositions of varying molecular weights. The evaluation encompassed physiological and biochemical indices, histopathological alterations, mucosal barrier integrity, levels of inflammatory mediators, and the composition of the intestinal microbiota. This approach aimed to elucidate the pharmacological basis and potential role of PA in the treatment of UC.

DSS is a negatively charged sulfated polysaccharide that inhibits epithelial cell proliferation, disrupts the intestinal mucosal barrier, and induces the release of cytokines from nonspecific immune cells, which leads to colitis (Zafarmand et al., 2022). It is the most widely used and well-studied experimental model for inducing UC in mice. It is characterized by ulceration and granulocytic infiltration that affects the integrity of the mucosal barrier, and shares many similarities with human UC. Therefore, the use of DSS to induce UC in mouse models is highly feasible.

Herein, 438 active peptides were identified, and 1,326 molecule compounds were extracted from the PA, including phenols, flavonoids, amino acids, and their derivatives, using Nano-UPLC-MS/MS and UPLC-MS analysis. Among them, phenols, flavonoids, amino acids and their derivatives exhibited the highest percentage of peak area, and these components collectively constitute the pharmacodynamic material basis of PA for the treatment of UC by enhancing the body’s ability to resist oxidative stress, promoting tissue repair, exerting anti-inflammatory and analgesic effects, and inducing apoptosis in cancer cells.

*In vivo* experiments demonstrated that PA and its molecular weight graded fractions (<3, 3–10, >10 kDa) significantly ameliorated the core pathological phenotypes of DSS-induced UC mice, including weight loss, elevated disease activity index (DAI), shortened colonic swelling, bloody stools, and diarrhea symptoms. The pathological process of UC is closely related to the imbalance of cytokine networks. In this study, we observed significant elevations in pro-inflammatory mediators, including tumor necrosis factor-α (TNF-α), interleukin-6 (IL-6), interleukin-17A (IL-17A), interferon-gamma (INF-γ), myeloperoxidase (MPO), and inducible nitric oxide synthase (iNOS), within the colonic tissues of mice induced with dextran sulfate sodium (DSS). Conversely, anti-inflammatory cytokines such as interleukin-4 (IL-4) and interleukin-10 (IL-10) were suppressed, mirroring the intestinal barrier damage observed in clinical UC patients (Jiang et al., 2021). PA and its molecular weight fractions (<3, 3–10, > 10 kDa) were effective in restoring cytokine balance. Notably, the complete PA exhibited superior anti-inflammatory properties compared to its components, suggesting that there may be synergistic anti-inflammatory effects among the components. Secondly, PA plays a crucial role in the repair of colonic mucosal tissue. It markedly enhances the proliferation of cuprocytes and stimulates mucin secretion, thereby facilitating the re-establishment of the protective mucus layer (Glover et al., 2022; Zheng et al., 2022). PA prevents the degradation of crypt structure, expedites the differentiation of crypt cells into mature epithelial cells (Yan et al., 2022), and mitigates inflammation-induced mucus layer loss and epithelial permeability abnormalities (Parikh et al., 2019). These findings indicate that PA supports crypt cell differentiation and cuprocyte function, counteracting the impaired regeneration and mucus loss associated with ulcerative colitis UC. In addition, PA treatment significantly reduces pro-inflammatory mediators (TLR4, MyD88, and NF-κB-p65), thereby inhibiting the inflammatory cascade and its potential tumorigenic transformation (Ke et al., 2023). It also enhances the expression of tight junction proteins (occludin-1 and zoster occludin-1), repairing increased intestinal epithelial permeability caused by protein deficiency (Li et al., 2023c). This blockade of the inflammatory factor cascade mediated by them, together with the enhancement of tight junction protein expression to deter pathogenic bacteria invasion, ultimately realizes the dual regulation of anti-inflammatory-barrier repair.

PA was demonstrated to effectively mitigate DSS-induced UC in mice by restoring gut microbiota dysbiosis, as determined through 16S rRNA sequencing analysis. This finding aligns with the recognized role of gut microbial imbalance in the pathogenesis of UC, which involves impairments in barrier function, immunomodulation, and metabolism. In the DSS-induced mouse model of UC, alterations in microbial composition and structure were observed. Analyses of alpha diversity, such as the increased Simpson’s index, and beta Diversity analyses (PCoA, NMDS) both indicated that PA administration enhanced microbial similarity and abundance across treatment groups. This resulted in a shift in the overall community structure towards that of the healthy control group. At the phylum level, PA demonstrated efficacy in reversing the dysbiosis characteristic of UC by reducing the aberrant enrichment of pro-inflammatory-associated phyla such as *Desulfobacterota*, *Actinobacteriota*, and *Verrucomicrobiota*, while correcting the depleted state of *Proteobacteria*. This modulation is closely linked to the inhibition of pro-inflammatory factor release (Hu et al., 2023; Knox et al., 2019; Zhu et al., 2022). At the family and genus level, PA selectively inhibits the amplification-mediated increase in epithelial permeability caused by the pathogenic bacterium *Desulfovibrionaceae* (Cao et al., 2024; Xiao et al., 2021)and mitigates the exacerbation of chronic inflammation associated with *Erysipelotrichaceae* (Kaakoush, 2015). Concurrently, it facilitates the re-establishment of the mucus barrier driven by *Akkermansiaceae* (Cheng et al., 2023a).

Subsequent LEfSe analysis identified UC-significant biomarkers, including *Desulfovibrio*, whose abundance was reduced following PA treatment. Notably, the modulation potency of the intact PA on the bacterial population was significantly superior to that of individual molecular weight components, indicating that multicomponent synergism is fundamental to its therapeutic efficacy. Furthermore, the relationship between cytokine levels and gut microbiota was examined at the genus level. The colony remodeling effects of PA were found to directly regulate intestinal immune homeostasis. Specifically, the abundance of pathogenic bacteria such as *Desulfovibrio* was significantly and positively correlated with proinflammatory factors INF-γ, IL-17A, and INOS. Their increased colonization may lead to antigen exposure and upregulation of the TLR4, MyD88, and NF-κB-p65 inflammatory mediators, which subsequently drive CD4^+^ T cells to differentiate toward the Th1/Th17 subtypes, thereby eliciting a cascade of inflammatory factors (Ma et al., 2018). Secondly, the proliferation of the probiotic *Psychrobacter* induced by PA is positively associated with the anti-inflammatory cytokines IL-4 and IL-10, which mitigate the immune response by inhibiting interactions between pathogens and epithelial cells. This bidirectional regulation of “pathogen inhibition and probiotic activation” ultimately restores intestinal micro-ecological balance and addresses the pathological processes of UC at their source (Wang et al., 2024). Furthermore, analysis using the Kyoto Encyclopedia of Genes and Genomes (KEGG) pathway revealed that PA ameliorates UC by influencing microbial functions related to secondary metabolite synthesis, amino acid biosynthesis, and glycolysis. This not only provides an innovative mechanistic explanation for the treatment of UC with PA but also establishes a theoretical foundation for colitis treatment through colony modulation.

## 5 Conclusion

In this study, we demonstrated that PA significantly ameliorated the pathological processes associated with DSS-induced UC. This was evidenced by the effective alleviation of core symptoms such as weight loss, shortened colon swelling, and the presence of blood in the stool. Furthermore, PA facilitated the reconstruction of the intestinal mucosal barrier function by restoring the balance between pro-inflammatory and anti-inflammatory factors, inhibiting the inflammatory mediators TLR4, MyD88, and NF-κB-p65, and enhancing the expression of tight junction proteins, including occludin-1 and ZO-1. Finally, PA reversed the enrichment of pathogenic bacteria by remodeling the intestinal flora, specifically *Desulfovibrio*, and activated mucosal barrier repair through *Akkermansiaceae*. This study elucidates the role of PA in UC treatment via the remodeling of the “flora-immunity-barrier” axis and suggests the therapeutic potential of the medicinal insect *Periplaneta americana* L. for UC treatment, offering insights into future therapeutic directions.

## 6 Limitations of the study

Our study is subject to three notable limitations. Firstly, while we observed a significant downregulation of TLR4, MyD88, and NF-κB p65 expression following PA treatment, the causal necessity of this signaling pathway was not functionally validated by knockout models or drug inhibition experiments. This limitation precludes us from definitively asserting whether the TLR4/NF-κB signaling pathway is essential for the efficacy of PA. Secondly, although it was demonstrated that PA induces the restoration of microbial diversity and inhibits pathogenic genera such as *Desulfovibrio*/*Dubosiella*, we did not conduct experiments involving fecal microbiota transplantation (FMT) from PA-treated mice into a colitis model. Consequently, direct evidence supporting the role of gut flora as a major mediator of treatment efficacy remains absent. Thirdly, this study did not systematically identify the chemical composition of different molecular weight fractions of PA after separation and fractionation, making it impossible to precisely associate specific components with their biological effects.

These limitations stem primarily from experimental time and resource allocation constraints. However, it is now established that intestinal immunotherapy will be a specific direction for future research, and future work will perform humanized fecal microbiota transplantation (FMT), whereby the microbiota of PA-treated mice is transplanted into a model of aseptic colitis, followed by polyomics integration to identify the key species that mediate therapeutic effects. Simultaneously, conducting detailed chemical characterization of the components in each molecular weight fraction of PA to clarify their pharmacological basis.

## Data Availability

The original contributions presented in the study are publicly available. This data can be found here: https://www.ncbi.nlm.nih.gov/sra/PRJNA1328476.
