# Supplementary material for: Active ingredients isolated from Periplaneta americana L. inhibit the inflammation of the colonic mucosa and regulate the gut microbiota in DSS-induced ulcerative colitis in mice
Source: Front Pharmacol. 2025 Sep 19;16:1615989. doi: 10.3389/fphar.2025.1615989 (PMC12492031; doi:10.3389/fphar.2025.1615989)
Supplement: Supplementary file 3 [file Table5.docx]

Materials

| Name | Purity | Grade | Manufacturer |
| --- | --- | --- | --- |
| Acetonitrile (CAN) | ≥99.9% | LC-MS grade | Fisher Chemical |
| Formic acid (FA) | 98.0% | LC-MS grade | Sigma-Aldrich |
| Ammonium bicarbonate | ≥99.5% | LC-MS grade | Sigma-Aldrich |
| Dithiothreitol (DTT) | ≥99% | analytical grade | Sigma-Aldrich |
| Iodoacetamide (IAA) | ≥99% | analytical grade | Sigma-Aldrich |

Main Instrumentation

| Name | Manufacturer | Model number |
| --- | --- | --- |
| Ultra-High Performance Liquid Chromatography | Sciex | ExionLC AD |
| High-Sensitivity Mass Spectrometry | Sciex | Qtrap 6500+ |
| Ultrapure Water System | Merck Millipore | MZ D24 UV |
| Chromatography Column | Waters | ACQUITY UPLC HSS T3 1.8 *μ*m 2.1*100 mm |
| Nanoflow Liquid Chromatography | Thermo Fisher Scientific | Ultimate 3000 |
| ESI-Q-Orbitrap MS | Thermo Fisher Scientific | Q Exactive™ Hybrid Quadrupole-Orbitrap™ Mass Spectrometer |
| Centrifugal Evaporator | Eppendorf | Concentrator plus |
| Analytical Balance | Sartorius | Sartorius BP211d |
| Refrigerated High-Speed Centrifuge | Beckman Coulter | Microfuge 22R Centrifuge |
| Micro Centrifuge | Scilogex | D1008 |
| Vortex Mixer | Scilogex | MX-S |
