# Supplementary figures and images for: Active ingredients isolated from Periplaneta americana L. inhibit the inflammation of the colonic mucosa and regulate the gut microbiota in DSS-induced ulcerative colitis in mice
Source: Front Pharmacol. 2025 Sep 19;16:1615989. doi: 10.3389/fphar.2025.1615989 (PMC12492031; doi:10.3389/fphar.2025.1615989)

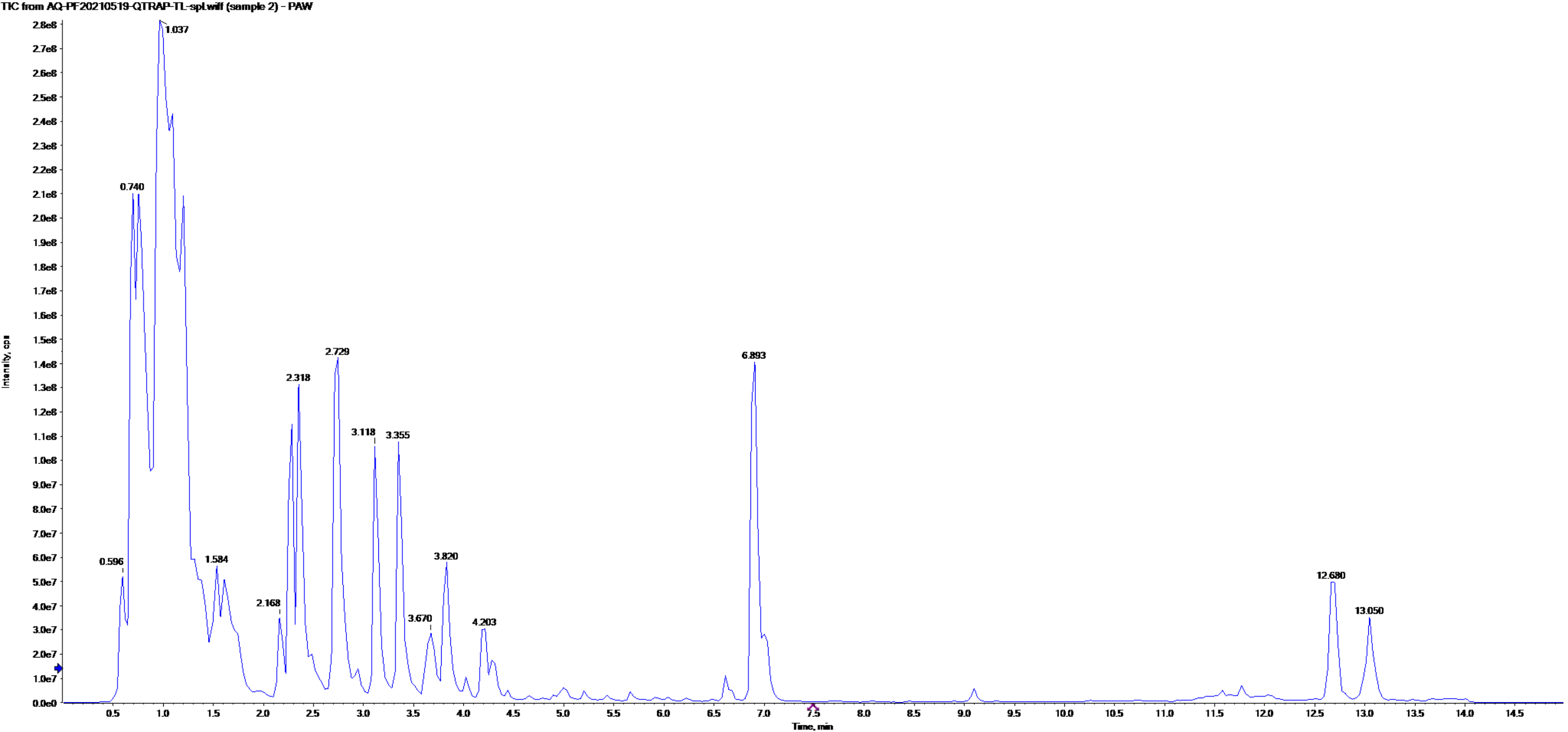

Supplement: Supplementary file 5 [file Image2.png]

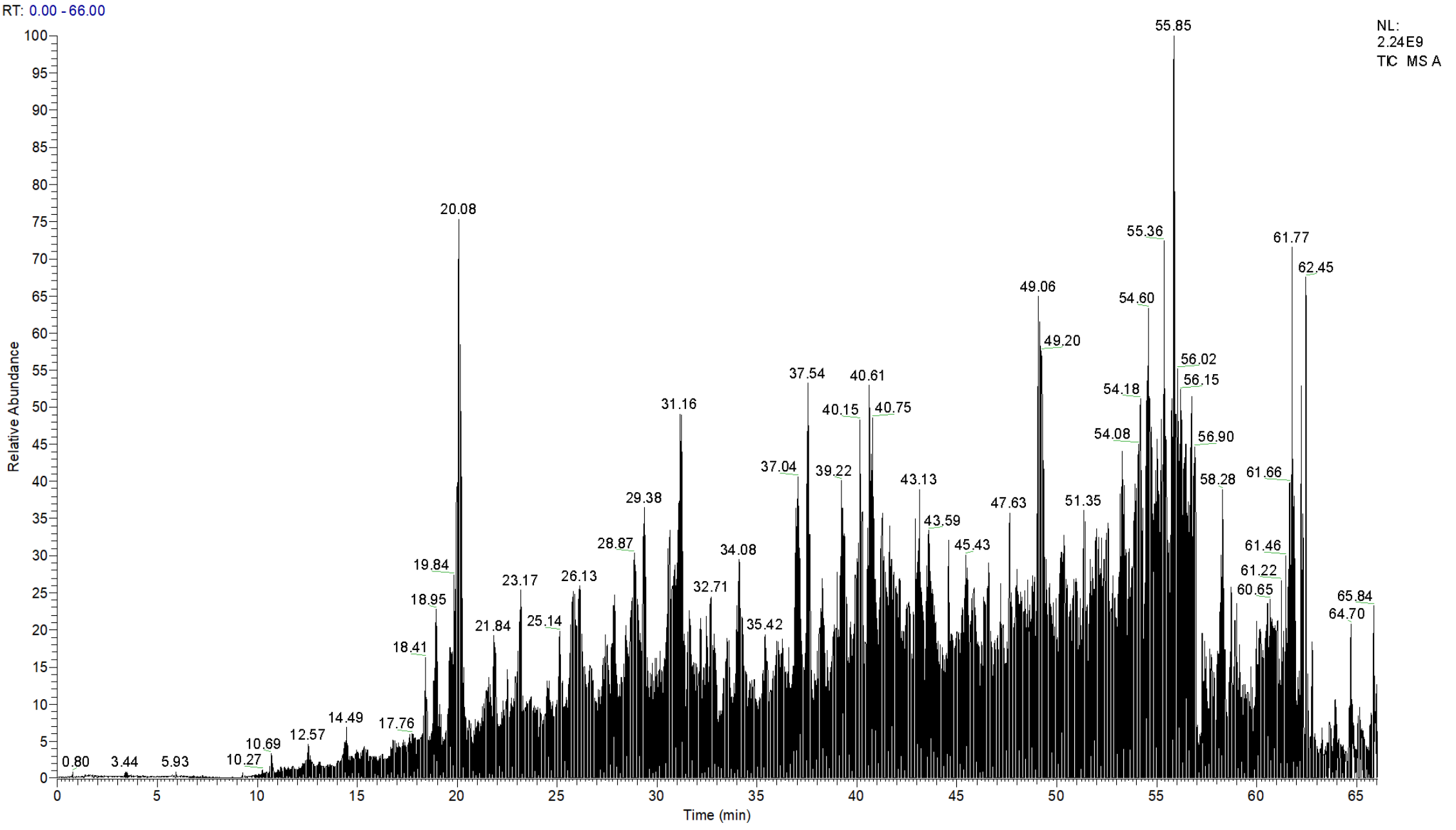

Supplement: Supplementary file 7 [file Image1.png]
